# 

**Published:** 1894-11

**Authors:** 


### CONTENTS:

ORIGINAL ARTICLES: PAGE

Neurectomy as a Practical Operation. By S. J. J. Harger, V.M.D. .... 365
Thirty-first Annual Convention U. S. Veterinary Medical Association :—
Report of Committee on Intelligence and Education .... 382
Report of Publication Committee .... 391
Report of Committee on Diseases .... 392
Report of Committee on Revision of Constitution and By-Laws .... 396
Report of Committee on Incorporation .... 398
Report of Committee on Army Legislation .... 401
Report of Committee on Nomenclature of Hog Cholera and Swine Disease .... 403
Secretary's Report .... 404
The Anatomy of the Large American Fluke (*Fasciola Magna*). By Charles Wardell Stiles, Ph.D .... 407

EDITORIAL:

Blood Serum Therapeutics .... 418
Embargo on American Cattle .... 418
Tuberculosis .... 419
How Might We Best Spend the Winter Sessions of our Association .... 420

SOCIETY PROCEEDINGS:

Thirty-first Annual Meeting of the United States Veterinary Medical Association .... 424
Missouri State Veterinary Medical Association .... 435
Keystone Veterinary Medical Association .... 436

